# Standardization and Quality Assessment Under the Perspective of Automated Computer-Assisted HEp-2 Immunofluorescence Assay Systems

**DOI:** 10.3389/fimmu.2021.638863

**Published:** 2021-02-25

**Authors:** Luigi Cinquanta, Nicola Bizzaro, Giampaola Pesce

**Affiliations:** ^1^ Laboratorio di Patologia Clinica, IRCCS S.D.N., Napoli, Italy; ^2^ Laboratorio di Patologia Clinica, Ospedale San Antonio, Tolmezzo—Azienda Sanitaria Universitaria Integrata di Udine, Udine, Italy; ^3^ Laboratorio Diagnostico di Autoimmunologia, IRCCS Ospedale Policlinico San Martino, Genova, Italy; ^4^ Dipartimento di Medicina Interna e Specialità Mediche (DIMI), Università Degli Studi di Genova, Genova, Italy

**Keywords:** harmonization, standardization, anti-nuclear antibodies, computer-assisted systems, immunofluorescence, automation

## Abstract

The recent availability of automated computer-assisted diagnosis (CAD) systems for the reading and interpretation of the anti-nuclear antibody (ANA) test performed with the indirect immunofluorescence (IIF) method on HEp-2 cells, has improved the reproducibility of the results and initiated a process of harmonization of this test. Furthermore, CAD systems provide quantitative expression of fluorescence intensity, allowing the introduction of objective quality control procedures to the monitoring of the entire process. The calibration of the reading systems and the automated image interpretation are essential prerequisites for obtaining reproducible and harmonized IIF test results and form the basis for standardization, regardless of the computer algorithms used in the different systems. The use of automated CAD systems, facilitating control procedures, represents a step forward for the quality certification of the laboratory.

## Introduction

The indirect immunofluorescence (IIF) assay on HEp-2 cells is considered the reference method for the screening of anti-nuclear antibodies (ANA) and plays a central role in the diagnosis of autoimmune rheumatic diseases. Its high diagnostic sensitivity allows the detection of over 30 different fluorescence patterns, corresponding to as many autoantibody specificities (1–4). However, the HEp-2 IIF method is currently limited by a low level of harmonization. Major drawbacks are high intra and inter-laboratory variability, semiquantitative expression of results and lack of specificity. The method is also time consuming and has a long turn-around-time (5–8). It was also pointed out that the high variability of the method jeopardizes the selection of patients to be included in clinical trials for the evaluation of therapeutic protocols (9). The main critical issues related to the search of ANA by HEp-2 IIF are shown in **Table 1**.

**Table 1:** Main issues in the standardization of the ANA HEp-2 immunofluorescence assay.

BIOLOGICAL FACTORS	VARIABLES	AFFECTION
HEp2 cell strain	Growth rate, antigenic distribution	Sensitivity and pattern recognition
Culture conditions	Medium, drugs (antibiotics), time, temperature	Antigen expression (sensitivity)
Slides processing	Different fixatives (alcohol/acetone solution, pure acetone, etc)	Sensitivity, specificity, stability
Conjugates	Isotype, species, type of target, purification method, fluorochrome, fluorescein/protein ratio, concentration, anti-folding	Sensitivity, specificity
**PROCEDURAL FACTORS**	**VARIABLES**	**AFFECTION**
Samples	Collection and storage temperature, freeze-thawing cycles, interfering factors (serum indices)	Repeatability and reproducibility
Preparation of the slides for reading	Manual vs automated, traceability	Repeatability and reproducibility
Microscope	LED vs. mercury lamp, optical quality, camera sensitivity	Sensitivity
Image interpretation	Expertise, training, computer assisted	Diagnostic capability
Cut-off verification	Collection of sera classified by clinical criteria, lack of reference sera	Diagnostic capability
**DECISION FACTORS**	**VARIABLES**	**AFFECTION**
Starting dilution	Diverging recommendations, differences in ethnicity and target populations	Diagnostic capability
Pattern nomenclature	Ambiguous descriptions, different names for the same antibody pattern	Reproducibility
Diagnostic strategy	Choice of the commercial method, diverse diagnostic algorithms, pre-test probability	Reproducibility and diagnostic efficacy
Reports	Non suitable requests, diverse information, limited lab-clinician communication	Diagnostic efficacy
Guidelines & recommendations	Diverging criteria, insufficient diffusion, limited implementation	Reproducibility

Probably the most important cause of variability in the detection of HEp-2 IIF ANA is represented by the subjectivity in titer and pattern interpretation, even when the reading is performed by expert personnel (10, 11). In this regard, external quality assessment (EQA) schemes have highlighted a significant discrepancy of the results, especially for samples with a cytoplasmic pattern and in the assessment of the antibody titer which, in some cases, may differ by more than two dilutions (12–14).

Other causes of variability are inherent in the reagents used. Differences in the HEp-2 substrate supplied by the various manufacturers mainly related to the growth time of cell cultures and the methods of cell fixation, are an important source of discrepancy (15, 16). The different substrates of HEp-2 cells available on the market significantly determine the non-uniform accuracy of the various diagnostic kits, not only in terms of overall sensitivity but also as regards the ability to detect autoantibodies directed against some antigenic specificities (17).

Another critical issue is the choice of the initial dilution of the screening test, which is directly linked to the diagnostic specificity of the method. There is now sufficient agreement that the threshold cutoff for ANA should no longer be fixed at 1:40. Accumulated evidence has made clear that the best compromise between sensitivity and specificity of the ANA test be at least 1:80. Furthermore, the choice of 1:80 as the best screening dilution is consistent with the results obtained by Tan et al. (18) on more than 22,000 healthy individuals, showing that this titer corresponds to the 95%ile of healthy controls, as recommended by the EASI group (4) and various national guidelines (19–21). The new classification criteria for systemic lupus erythematosus also recommend a screening dilution of 1:80 (22).

## ANA HEp-2 IIF Detected by Automated Computer-Assisted Systems

**Figure 1:**
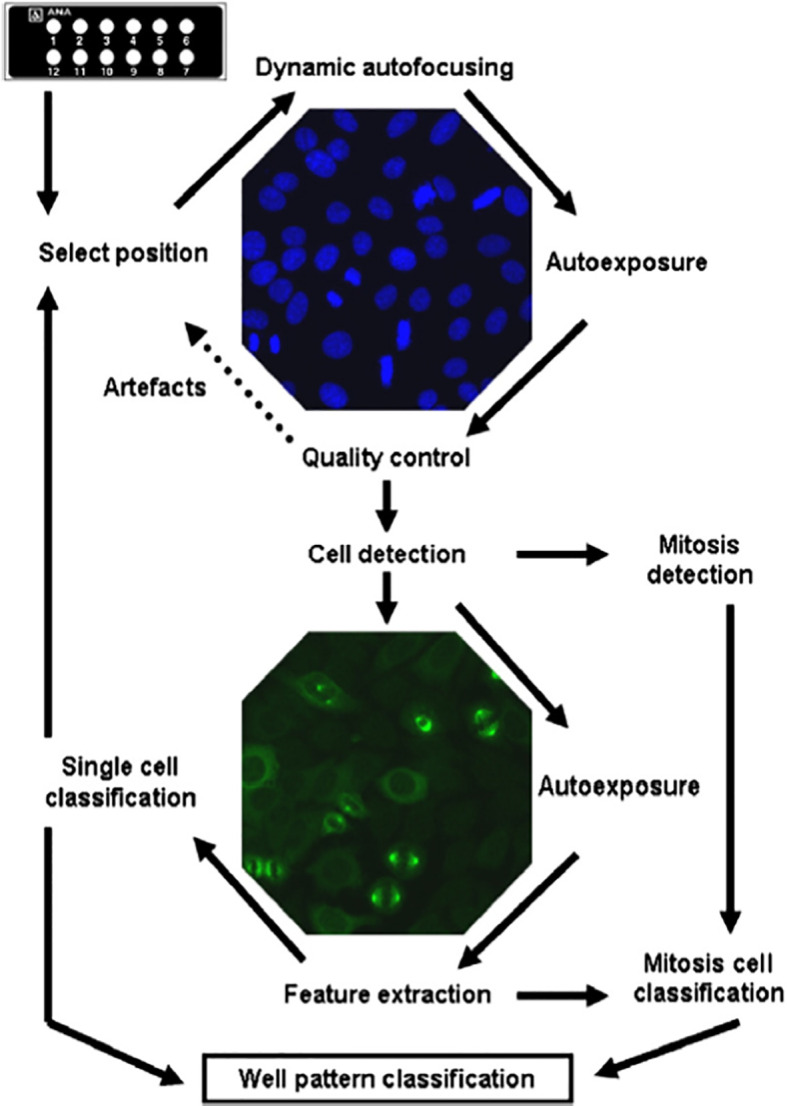
Complete processing cycle of automated HEp-2 cells assay reading by Aklides system (reproduced from Hiemann R, et al. Challenges of automated screening and differentiation of non-organ specific autoantibodies on HEp-2 cells. Autoimmunity Rev 2009; 9:17-22) (29).

In an attempt to overcome some of the disadvantages of manual HEp-2 IIF tests, the biomedical industry, in addition to the development of fully automatic slide processors to standardize the pre-analytical phase, has developed computer-aided diagnosis (CAD) technologies to digitalize ANA HEp-2 IIF analysis (23–28). These systems arise from the combination of various hardware modules which, using software based on complex mathematical schemes and algorithms, are able to acquire, analyze and store the images in a fully automated way (29, 30) (**Figure 1**).

One of the most important advantages of CAD systems is that they offer a more standardized, automated quantitative reading of the fluorescence signal, translated into system specific fluorescence intensity (FI) measures. In a meta-analysis that compared the diagnostic accuracy of CAD systems with that of manual methods for HEp-2 IIF, CAD systems showed overall greater agreement in the estimation of results and less variability in the definition of antibody levels compared to manual methods. Furthermore, in the screening of systemic autoimmune diseases, automated methods have proved more sensitive than manual ones (31).

Through the digitization of the images, CAD systems aim not only to determine the reduction of the variability of the HEp-2 IIF tests, minimizing the subjectivity of the interpretation of the fluorescence patterns (31–34), but also to increase the productivity of the laboratory, eliminate the use of the darkroom, allow the archiving of images for future check, ensure sample traceability through the barcode, and electronic data transmission (35).

However, despite the obvious improvements in the harmonization of results, given that these new computerized systems use HEp-2 cells, they still suffer from some of the inherent problems of the manual HEp-2 IIF method. Furthermore, like all analytical systems produced by various manufacturers, CAD systems differ in DNA counterstaining (DAPI, propidium iodide, none), substrate composition, run time, number of microscopic fields processed, type of recognized HEp-2 IIF patterns and the interpretative software of the acquired images (24, 27).

The nature of the light sources and the specifications of the microscope optics may also be a cause of inconsistency (36–38). Differences in the technical specifications of the light emitting devices, filters and lenses, can lead to a high variability in the intensity of the excitation light used in CAD systems. In these automated systems the drop in intensity of the LED lamp, the degradation of the camera sensor, the whitening of the fluorescent filter, the misalignment of the light path, may have an impact on the intensity of the emitted fluorescence (39, 40).

In a study involving 31 Belgian laboratories using different automated CAD systems, reproducibility of results and sufficient accuracy in estimating dilution was observed in a limited number of laboratories, while the overall results indicated that significant variability persisted in the detection of ANA. It should be noted that not only variability was found between the results of automated HEp-2 IIF assays from different manufacturers but also between those obtained from instruments of the same manufacturer (41).

Finally, as regards the interpretation of the pattern, it cannot be overlooked that automated CAD systems are currently able to recognize only some fluorescence patterns, mainly the homogeneous, speckled, nucleolar, centromeric, nuclear dots and cytoplasmic. Hence, visual reading by the operator at the monitor is still considered essential in order to assign the pattern and for subsequent reporting. To perform the diagnosis by looking at digital images on a workstation monitor allows the specialists to better concentrate on sample examination, e.g. to observe carefully fine details without take care of photobleaching effects. The observers were initially not accustomed to diagnose the sample using the workstation monitor, while they were well skilled in carrying out the diagnosis at the microscope. Therefore, the results on digital image classification could potentially remarkably improve as the expertise with this kind of diagnostic procedure increases and even the less frequent patterns, not recognized today by CAD systems, can be identified more accurately by the specialist.

## Standardization/Harmonization of Automated ANA HEp-2 IIF Assays

The standardization of autoantibody tests is generally considered to be among the most challenging in the context of *in vitro* diagnostics (42). The main reason is that measurands, i.e. antibodies, are made up of a highly variable mixture of different molecules in terms of epitope recognition, degree and type of glycosylation, isotypes and subclass distribution, and degree of avidity (43, 44).

Standardization can be defined as the process of implementing a standard preparation capable of maximizing the compatibility, even quantitative, of test results and possibly achieving their uniformity. Harmonization, on the other hand, can be defined as mediation between different measurements obtained with different methods and procedures to make them mutually compatible. Harmonization is generally reached by agreement between the parties concerned and is formalized in recommendations and/or guidelines (45, 46).

Therefore, if standardization in autoimmunology is a very difficult goal to achieve and will likely take a long time, the use of automated CAD systems is expected to improve right away the harmonization of the reading of HEp-2 IIF. In particular, two important benefits are expected: greater agreement in discriminating between positive and negative ANA samples, and lower imprecision in the definition of antibody titer/concentration. Currently available data show that the concordance between conventional HEp-2 IIF interpretation and automated systems in correctly expressing positive and negative results varies between 92% and 99% (24, 25, 30, 47, 48). In samples with ANA tests that are clearly negative or highly positive, CAD systems achieve a degree of accuracy close to 100% (49). The greater reproducibility of the results provided by the new automatic methods was demonstrated in a study that compared the analytical imprecision of six CAD systems vs. the manual HEp-2 IIF method. The mean coefficient of variation (CV) was 12% for the CAD vs. 39% for manual IIF (24).

A further contribution to the harmonization of the process concerns the choice of the cutoff titer, which is fundamental for a correct classification of the samples as positive or negative. While it would be recommended for each laboratory to determine its own screening dilution for the local population to distinguish healthy and diseased states, in practice, this procedure is not followed by the vast majority of laboratories because there is a high consensus in the literature that the titer of 1:80 can be considered the best compromise between diagnostic sensitivity and specificity (21, 36, 50–52). Furthermore, since the titer 1:80 is the screening dilution adopted by all manufacturers of CAD systems for automated reading and interpretation of ANA (24), this methodological approach represents a first and concrete step to achieve the harmonization of ANA HEp-2 IIF results. Indeed, if different laboratories should adopt different cutoffs, this would diminish comparability of results and therefore decrease harmonization.

However, given that the fluorescence signal is strongly dependent on the antibody pattern because of the variable concentration and cell distribution of the self-antigens, different staining patterns are characterized by a different FI mean for the same end-point titer. This issue has been faced by manufacturers of CAD systems developing built-in calibration curves for each one of the most common ANA patterns. To prove this relationship, Carbone et al. calculated R2 on a single fitted lines plot obtained by plotting FI as a function of dilution factor for whole serum series and for 10 different antibody patterns. Regression analysis showed a close relationship between FI and titer dilution for each pattern (53).

Since an accurate extrapolation of antibody titer based on fluorescence intensity is not possible with only a single screening dilution and this method cannot be applied to mixed ANA patterns, Won (54) proposed to use the line slope titration (LST) method using at least two distant point dilutions (i.e., 1:80 and 1:320) which would enable a better prediction of end-point titers based on the measured FI and evaluate possible prozone effects avoiding serial dilutions. To this end, an interfacing middleware to calculate the endpoint titer using LST should be implemented between automated CAD software and the laboratory information system (54).

While the advent of CAD systems has already contributed to improving ANA HEp-2 IIF assay, for a wider harmonization of the test, other aspects must be considered. Uniform terminology is also needed in the description of the HEp-2 IIF patterns. In a context characterized by the absence of a universally accepted nomenclature and by a substantial subjectivity in the interpretation of fluorescence patterns, the International Consensus on ANA Patterns (ICAP) had the merit of laying the foundations for the harmonization of the terminology, of providing guidelines for the interpretation of test results and to indicate the reporting format (55–57). ICAP has also defined the clinical relevance of the distinct HEp-2 IIF patterns, also indicating the appropriate use of in-depth tests, and has promoted the translation of the information content into multiple languages, to facilitate the unambiguous diffusion of the classification system in different countries of the world (58).

Reporting the ANA test result as positive or negative in the presence of cytoplasmic and mitotic patterns (CMP) is still a controversial topic (22, 59). However, although there is still no general consensus, given that CMP are observable in the HEp-2 IIF assay along with the nuclear patterns, some guidelines have recommended that CMP should be included in the ANA positive definition (4, 60–62).

## Quality Assessement

In addition to automated procedures for the validation of the analytical process, the HEp-2 IIF CAD systems, due to their ability to report FI quantitative results, allow the introduction of quality control (QC) procedures using objective acceptance criteria for each analytical session (7). Quality assurance can be based on daily monitoring of the measured FI values ​​for positive and negative QC samples, evaluated with the traditional Westgard rules, 1_2CV_ as the alarm limit and 1_3CV_ as the limit to reject the series (63, 64). In this regard, however, it has been pointed out that the use of only internal quality control (iQC) materials provided by the manufacturers of the diagnostic kits cannot highlight all possible analytical errors (65) because iQC samples in the diagnostic kit are usually ready-to-use and do not require pre-dilution like routine patient samples. In addition, according to van der Bremt et al, the effect of some apparently trivial variables (i.e., the efficiency of the conjugate) is not evident using iQC samples associated with the highest FI values ​​but only with those with FI values ​​around the positivity limit (33). For a more adequate quality assurance, the introduction of additional quality indicators has been proposed, such as the evaluation of the median of the results of the FI of iQC samples obtained from pooled patient sera, and the monitoring of the percentage of ANA IIF positive results in the analytic session (65, 66).

**Figure 2:**
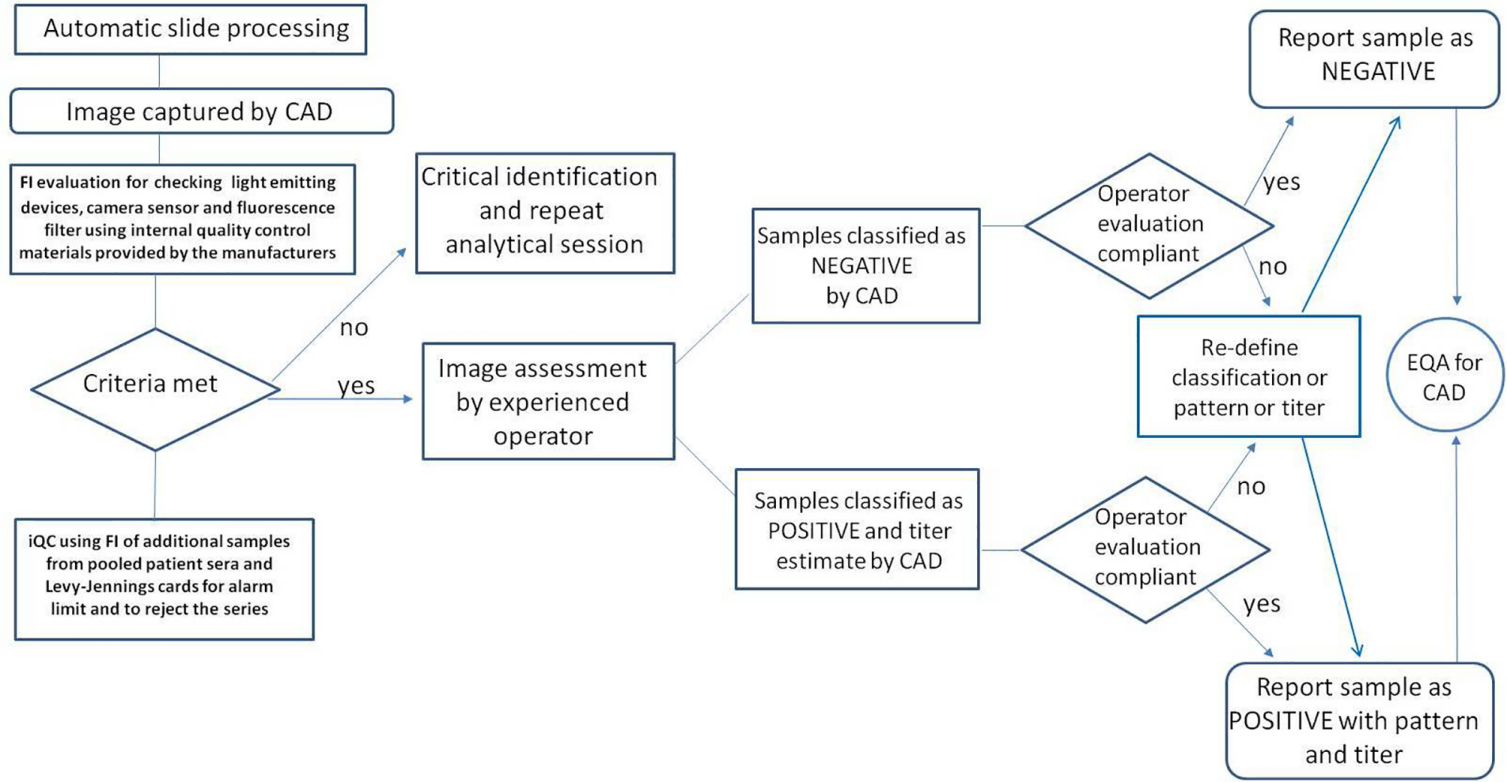
Steps related to quality control and interpretation of the results using the automated CAD procedure for the determination of ANA in indirect immunofluorescence.

Subsequently, a wider participation in EQA programs will be required to monitor the performance of each CAD system in order to comprehensively address the harmonization of the HEp-2 IIF test (33). In this context, it is important that EQA programs are dedicated to CAD assays or at least evaluated separately from manual methods (**Figure 2**).

Furthermore, integrating FI based iQC charts into the routine ANA IIF workflow offers a solution to current shortcomings of autoimmune laboratory testing in achieving ISO 15189 accreditation and could bring this branch of autoimmunity closer to other immunometric assays and their well-established rules (64, 65, 67–69). To this end, it is the responsibility of the laboratory autoimmunologist to evaluate and control all the variables that have a potential impact on the total processing of the HEp-2 IIF test (70, 71). In this context, neither pre-analytical variables such as the type and degree of suspected pathology underlying test request, nor analytical (errors in the washing or dispensing of reagents), or post-analytical ones (expression of results and introduction of interpretative notes in the report through the laboratory information system) should be neglected.

## Discussion and Future Perspectives

In recent years, technological evolution has allowed the development of solid phase assays (SPA) for the research of ANA, which have proved to be slightly less sensitive but more specific than the HEp-2 IIF method (either manual or automated). In turn, this has led many researchers to propose the association of a SPA method with HEp-2 IIF as the best strategy to increase the diagnostic efficiency of ANA research (72–77). Whatever the choice, whether performed alone or in combination with SPA methods, the HEp-2 IIF method will continue to play a central role in the diagnosis of autoimmune rheumatic diseases. For this reason, efforts to further improve the performance of the HEp-2 IIF method and the test standardization and harmonization process should not be abandoned or slowed down.

The development of more characterized standards and reference materials is the first step towards the standardization of autoantibody tests. Such reference materials should ideally be homogeneous, stable, traceable, switchable, safe, ethically obtained, available and, ideally, certified. A promising and concrete initiative underway by the International Federation of Clinical Chemistry and Laboratory Medicine (IFCC) Committee on Harmonisation of Autoantibody Testing aims at the preparation of serum pools with monospecific samples obtained from an adequate number of donors (78). Numerous variants of the same antibody will be included in the pool to minimize batch-to-batch differences. However, the complexity and variability of antigens, antibodies and analytical methods makes it unlikely that the introduction of antibody standards alone will completely solve all standardization problems. It is more likely that it will represent the beginning of the standardization process of the entire supply chain including not only the antibody but also the antigenic substrate and the analytical method.

It is necessary that the biomedical industry produces a further effort aimed both at expanding the spectrum of patterns that can be identified (for example the dense fine speckled) consistently with those classified by ICAP, and at the recognition of mixed patterns (35, 79). The implementation of the ICAP nomenclature, despite being already widespread, is believed to be only a first step towards the common goal of harmonizing the interpretation of HEp-2 IIF tests. According to a recent survey by Lisa Peterson et al. for US respondents, there is a need for further guidelines, consent documents, control/reference materials to promote the formation of the skills necessary to uniquely report the rarest and complex fluorescence patterns (80).

The electronic setting of each CAD system should be optimized in each operational reality, providing for the possibility of modifying the IF threshold value established by the manufacturer to classify the test as positive or negative, based on the state of efficiency of the individual components of the analytical instrumentation, so that the IF threshold value always corresponds to the titer of 1:80 chosen as the discriminant cutoff.

Finally, assigning the likelihood ratio (LR) value or post-test probability of disease to the HEp-2 IIF test result represents a new reporting approach in the field of ANA testing that can facilitate the clinical interpretation of test results and, by improving the comparability of the results from different analytical methods, contribute to harmonizing autoimmune laboratory reporting (81). The CAD systems, expressing the ANA test results quantitatively as FI values​ make the calculation of the LR easier, especially if the relationship between pre and post-test probability is represented graphically as a function of LR (62, 82).

## Conclusions

The standardization/harmonization of ANA tests is far from complete. A closer collaboration is necessary between autoimmunologists and the biomedical industry for the adjustment of diagnostic kits. The standardization process will be greatly accelerated when international standards and independent and certified calibrators are available and disseminated. The objectives are therefore to produce commutable materials that could be used as interim calibration material for autoantibody assays; to evaluate the impact of new reference material on the variability of autoantibody tests; and to identify areas where further harmonization would improve diagnostic accuracy. In this scenario, the international harmonization of diagnostic kits for HEp-2 IIF tests and the correct management of automated CAD systems for reading fluorescence preparations are the key points for the standardization of ANA research in immunofluorescence using HEp-2 cells.

## Author Contributions 

LC, NB, and GP drafted and revised the manuscript. All authors contributed to the article and approved the submitted version.

## Conflict of Interest

The authors declare that the research was conducted in the absence of any commercial or financial relationships that could be construed as a potential conflict of interest.
